# Maximizing the Impact of Training Initiatives for Health Professionals in Low-Income Countries: Frameworks, Challenges, and Best Practices

**DOI:** 10.1371/journal.pmed.1001840

**Published:** 2015-06-16

**Authors:** Corrado Cancedda, Paul E. Farmer, Vanessa Kerry, Tej Nuthulaganti, Kirstin W. Scott, Eric Goosby, Agnes Binagwaho

**Affiliations:** 1 Brigham and Women's Hospital, Boston, Massachusetts, United States of America; 2 Harvard Medical School, Boston, Massachusetts, United States of America; 3 Partners In Health, Boston, Massachusetts, United States of America; 4 Seed Global Health, Boston, Massachusetts, United States of America; 5 Massachusetts General Hospital, Boston, Massachusetts, United States of America; 6 Clinton Health Access Initiative, Boston, Massachusetts, United States of America; 7 Harvard University, Cambridge, Massachusetts, United States of America; 8 University of California San Francisco, San Francisco, California, United States of America; 9 Ministry of Health of Rwanda, Kigali, Rwanda; 10 Geisel School of Medicine—Dartmouth, Hanover, New Hampshire, United States of America

## Abstract

Corrado Cancedda and colleagues outline a framework for health professional training initiatives in low-income countries.

**Summary Points**

- Historically, the impact of many health professional training initiatives in low-income countries has been limited by narrow focus on a small set of diseases, inefficient utilization of donor funding, inadequate scale up, insufficient emphasis on the acquisition of practical skills, poor alignment with local priorities, and lack of coordination.
- Fortunately, several innovative training initiatives have emerged over the past five years in sub-Saharan Africa. This articles focuses on four initiatives funded by the United States government: the Medical Education Training Partnership Initiative (MEPI), the Nursing Training Partnership Initiative (NEPI), the Rwanda Human Resources for Health Program (HRH Program), and the Global Health Service Partnership (GHSP).
- The best practices adopted by these initiatives are: alignment to local priorities, country ownership, competency-based training, institutional capacity building, and the establishment of long-lasting partnerships with international stakeholders,
- Based on these best practices, we outline a framework for health professional training initiatives that can help better address the health workforce shortage in low-income countries.

## Introduction

### The Global Shortage of Health Professionals

The immense suffering taking place in West Africa due to the Ebola epidemic is a tragic and powerful example of an “acute on chronic” problem facing many low-income countries: the health workforce shortage [1].

Insufficient training capacity and the “brain drain” of health professionals from Africa are principal drivers of the current situation [1–3]. Health professional schools in low-income countries face notable limitations in physical space, equipment, curricula, training materials, faculty, administrative staff, and funding [4–7]. These limitations stifle efforts to expand the number and the diversity of training programs and to improve the quality of training. Simultaneously, practicing health professionals are often overwhelmed by the grinding work of delivering health services in under-supplied and over-crowded hospitals and clinics, inadequately compensated for their work, and demoralized by a lack of continuing professional development opportunities [1,3,8].

The health workforce shortage has negatively affected the response to the global HIV/AIDS epidemic, to the emerging threat of non-communicable diseases in sub-Saharan Africa, and most recently, to the Ebola epidemic in West Africa. To improve health outcomes globally, it is critical to increase the number and to diversify and strengthen the competencies of health professionals in low-income countries [1–4,9].

## Past and Current Efforts to Address This Shortage

**Fig 1:**
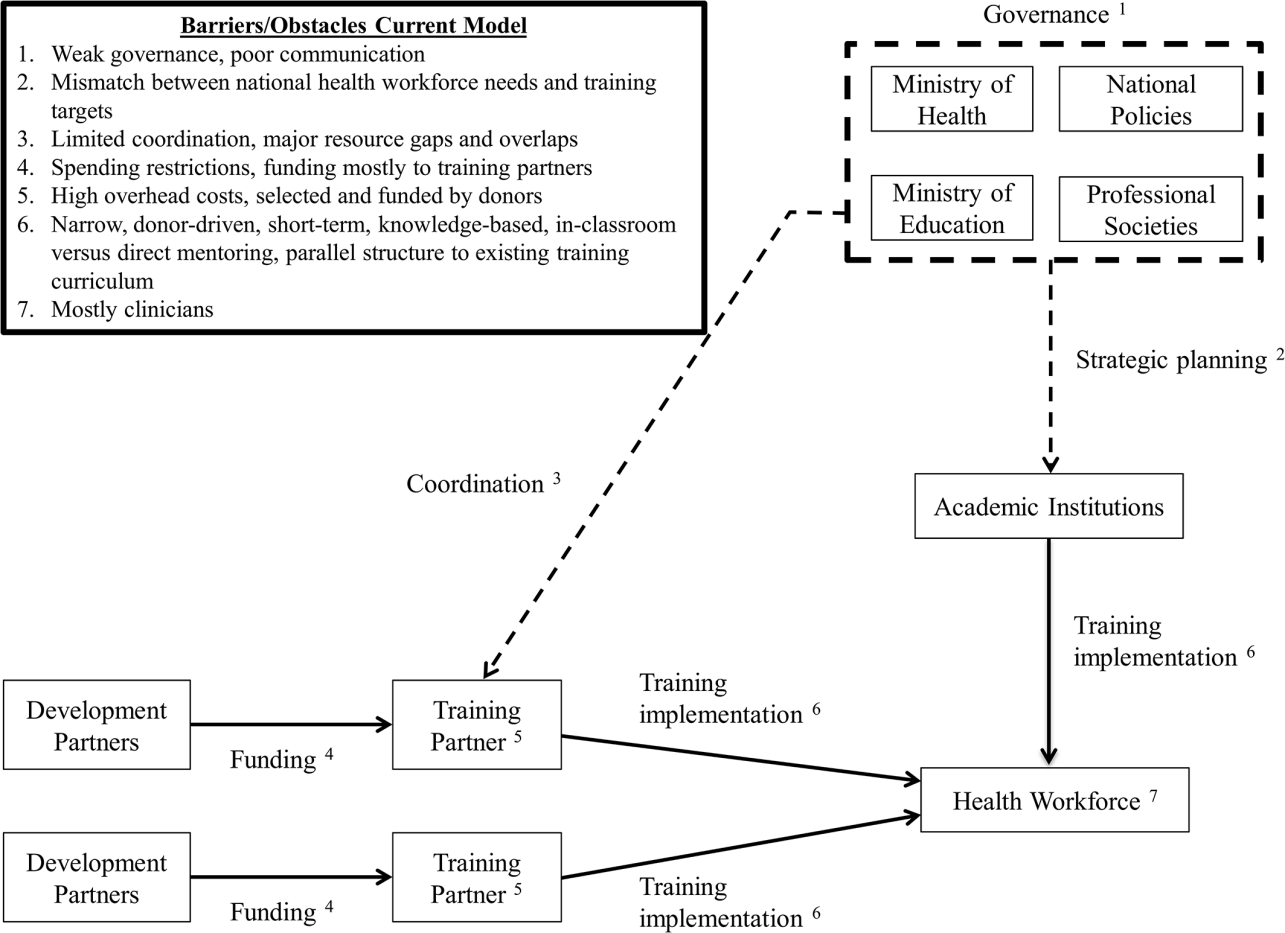
Current framework and practices for training initiatives aimed at increasing health professionals in low-income countries.

The number of health professional training initiatives in low-income countries has significantly increased over the past ten years [4,7,10,11]. A diverse range of internal (e.g., local governments and academic institutions) as well as external stakeholders have been involved in developing and implementing these initiatives [4,7,12]. External stakeholders have included both development partners (which contribute funding) and training partners (which contribute expertise and assist with training implementation) (Table 1). Especially in earlier years, the framework adopted by many of these training initiatives has led to less-than-ideal outcomes (Fig 1) [2,4,5,7,8,10,11].

**Table 1:** Current landscape: Examples of healthcare workforce development and training partners.

**Examples of Development Partners**
**Bilateral Development Agencies**
United States Agency for International Development
Norwegian Agency for Development Cooperation
Swedish International Development Cooperation Agency
United Kingdom Department For International Development
**National Research Institutes and Centers**
National Institute of Health
US Center for Disease Control and Prevention
**Multilateral Donors**
Regional Development Banks
The World Bank Group
**Global Health Initiatives**
United States President’s Emergency Plan for AIDS Relief
Global Fund to Fight AIDS, Tuberculosis, and Malaria
**Private Philanthropy**
Bill & Melinda Gates Foundation
ELMA Foundation
Rockefeller Foundation
Wellcome Trust
**Examples of Training Partners**
**Academic Institutions**
Academic Medical Centers
Medical Schools
Nursing Schools
Schools of Management
Schools of Public Health
**Non-Governmental Organizations**
Clinton Health Access Initiative
IntraHealth
JHPIEGO *(formerly Johns Hopkins Program for International Education in Gynecology and Obstetrics)*
Management Sciences for Health
Partners In Health
Seed Global Health
Volunteer Services Oversees

First, many of these initiatives have been primarily driven by the priorities of individual development and training partners and have often focused on a narrow set of diseases. Additionally, integration into national strategic plans (when they exist) or alignment with local priorities has often been marginal [2,4,5,10]. As a result, the same initiatives have rarely been brought to scale and have not addressed the health workforce shortage comprehensively [4,5,7,13,14].

Second, donor funding generally has come with many spending restrictions, which have prevented governments from utilizing the funds effectively, if at all [15,16]. In the past, many development partners have selected and directly funded training partners with limited input from local governments and local academic institutions [13]. These training partners often have spent a substantial proportion of funds on overhead rather than direct training costs [15,16]. Lastly, spending restrictions have often prevented training partners from investing in critical infrastructure and equipment within health professional schools and teaching hospitals that are necessary to create a strong teaching environment [13,14].

Third, many health professional training initiatives have prioritized mostly classroom teaching. Such ad hoc, short-term lectures and seminars have not been shown to effectively diversify the skills and strengthen the competencies of local health professionals [4,7,8,13,14]. Until recently, the competencies that allow different cadres of health professionals to work together as a team have been rarely addressed by curricula and training materials [9]. Furthermore, many initiatives have focused on training clinicians as opposed to other health professionals (e.g., health managers, community health workers, public health professionals, or researchers) [4,5]. The duration of these initiatives has been determined more by the arbitrary availability of funding and training expertise than by the time required for building local institutional capacity [4,7,8,13,14].

Lastly, governments and academic institutions in low-income countries face the overwhelming challenge of coordinating multiple health professional training initiatives, aligning them to national priorities, and integrating them with initiatives already being implemented in the country [2,4,5,13,14]. The challenge is further aggravated by the weak governance structures and lack of communication in many low-income countries among key policymaking entities, such as local ministries of health or health regulatory bodies and professional societies.

As a result of these limitations, low-income countries have been on the receiving end of a disorderly patchwork of small-scale, insufficient quality, short-term, and unsustainable health professional training initiatives that have focused only on a few diseases, created unnecessary gaps or overlaps in resources, and failed to help meet long-term national health workforce needs [2,4,5,7,8,14].

## New Models for Increasing Health Workforce Capacity

A number of innovative programs exist to address health workforce shortages globally, including the Cuban medical training model to address shortages in Pacific Island Countries; the MEDUNAM program between Finland, Namibia, and Mozambique; and Danish International Development Assistance (DANIDA) support of country-led national HRH plans in various countries [17,18]. We focus here on four health professional training initiatives funded entirely or partially by the US government (through the US President’s Emergency Plan for AIDS Relief [PEPFAR]) that have emerged over the past five years to address these shortages in sub-Saharan Africa. These US-based initiatives are the Medical Education Training Partnership Initiative (MEPI), the Nursing Training Partnership Initiative (NEPI), the Rwanda Human Resources for Health Program (HRH Program), and the Global Health Service Partnership (GHSP) (Table 2) [15,19–21]. Though they vary in both scale and scope, we believe their characteristics are useful to summarize in the context of this new framework for addressing health workforce shortages globally.

**Table 2:** Characteristics of new health professional training initiatives.

Name	Host Country or Countries	Budget^1^	Duration	Type (and Number) of Academic Institutions Supported in Host Countries	Main Health Professional Cadres Targeted^2^
Medical Education Partnership Initiative (MEPI)	Botswana, Ethiopia, Ghana, Kenya, Malawi, Mozambique, Nigeria, South Africa, Tanzania, Uganda, Zambia, Zimbabwe	US$130 million	5 years	Medical school (13), academic medical center (3), other academic institution (6)	Physicians (new graduates, specialists), nurses, other (pharmacy, dentistry, etc.), educators, researchers
Nursing Education Partnership Initiative (NEPI)	Democratic Republic of Congo, Ethiopia, Lesotho, Malawi, Zambia	US$33 million	5 years	Nursing schools (19)	Nurses and midwives, educators, researchers
Human Resources for Health Program (HRH)	Rwanda	US$170 million	8 years	Medical school (1), nursing school (5), dentistry school (1), scademic medical center (3), school of public health (1)	Physicians [new graduates (557) specialists (401)], nurses and midwives (2871), oral health professionals (302), managers and implementers (140)
Global Health Service Partnership (GHSP)	Malawi, Tanzania, Uganda	US$26.5 million^3^	Indeterminate	Medical school (2), nursing School (4), academic medical center (5), other academic institution (2)	Physicians (new graduates, specialists), nurses, midwives

^1^Funding for each program provided partially or in full by the US President’s Emergency Plan for AIDS Relief (PEPAR) Program; For more information for each training initiative, please visit: MEPI: http://www.pepfar.gov/partnerships/initiatives/mepi/index.htm; NEPI: http://www.pepfar.gov/partnerships/initiatives/nepi/; HRH: http://www.hrhconsortium.moh.gov.rw; GHSP: http://www.peacecorps.gov/volunteer/globalhealth/

^2^Projected number of trainees in parentheses

^3^For first six years

### Proposed New Framework and Best Practices for Training Initiatives in Low-Income Countries

**Fig 2:**
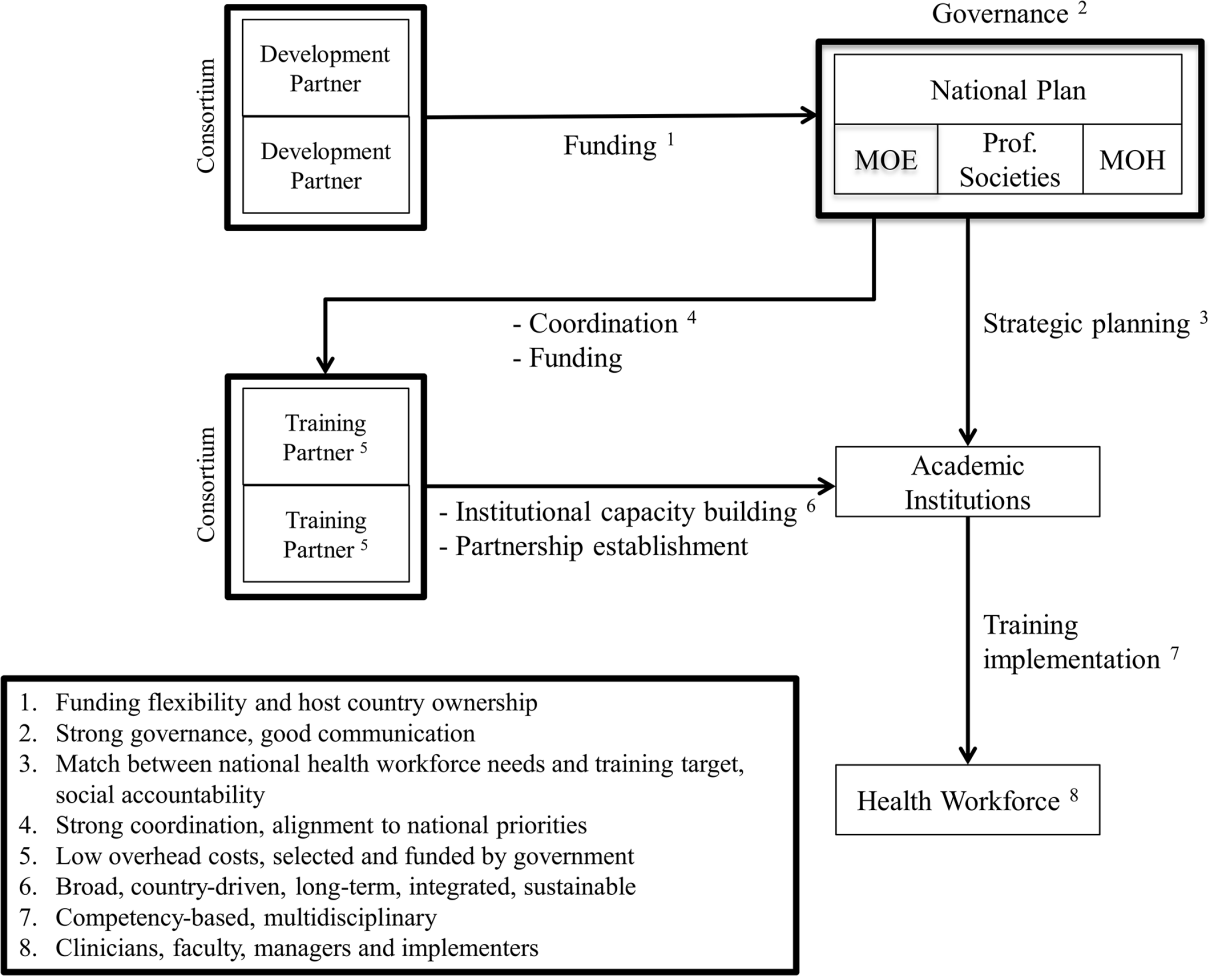
Proposed new framework and practices for training initiatives aimed at health professionals in low-income countries.

As contributing authors, we have played a critical role in the development and implementation of these training initiatives. In this article we outline a new framework that is informed by the following best practices, which we determined to be shared to a certain degree by all four training initiatives (Fig 2):

- Alignment to local priorities, joint planning, and coordination
- Funding flexibility and host country ownership
- Competency-based training and pedagogic innovation
- Institutional capacity building
- Sustainability strategy
- Establishment of long-lasting partnerships and communities of practice

Adoption of such a framework and best practices could inform future training and development partners as they seek to help local governments and academic institutions build a diverse, large, and competent health workforce in low-income countries.

### Alignment to Local Priorities, Joint Planning, and Coordination

Health professional training initiatives should be guided primarily by the national strategic plans of low-income countries rather than by the priorities of individual development and training partners [2,4,5,13–15]. When strategic plans do not exist, local and international stakeholders should engage in extensive negotiations and joint planning before initiating implementation. Governance bodies, ideally embedded within local governments or academic institutions, should be established to harmonize individual contributions in funding and training expertise. Lastly, the scale of training initiatives needs to be commensurate to the needs of low-income countries in order to bridge at least some of the gaps between both the number and the type of available and needed health professionals. Such an approach is consistent with the guiding principles espoused by the Paris Declaration on Aid Effectiveness, including harmonization and ownership [22].

For example, MEPI and NEPI have established two transnational coordinating centers (one at George Washington University and one at the African Centre for Global Health and Social Transformation) and separate governance bodies, advisory groups, or academic consortia within each host country, at which Ministries of Health, Ministries of Education, academic institutions (US and local), and health professional associations align priorities and agree on implementation strategies [23–25]. Similarly, the HRH Program in Rwanda established a steering committee and four technical working groups—consisting of representatives from the Ministry of Health, Ministry of Education, and US Agency for International Development (USAID)—to facilitate joint planning between the local health and education sectors. After the start-up phase, when seconded staff from Clinton Health Access Initiative provided strategic planning and management support, the HRH Program is now managed entirely by Ministry of Health staff. Moreover, the 25 participating US academic institutions have organized into a consortium to promote greater coordination and collaboration [15]. Lastly, the GHSP leadership has collaborated directly with the Ministry of Health, Ministry of Education, and medical and nursing councils of Tanzania, Malawi, and Uganda (and their partner academic institutions) to align training targets and national health workforce needs and to select an adequate number of training sites [21].

### Funding Flexibility and Country Ownership

Funds from development partners should come with fewer spending restrictions [13–16,26]. Also, they should allow low-income countries to adopt a “diagonal” approach to development and strengthen institutions and systems [13,26–28]. For example, many sub-Saharan African countries participating in MEPI, as well as Rwanda (as the host-country for the HRH Program), have utilized funds for infrastructure and equipment within health professional schools and teaching hospitals [15,25,29]. Moreover, all academic institutions participating in the HRH Program have agreed to charge overhead costs at less than 10% [15], while Seed Global Health (as partner non-governmental organization [NGO]) charges no overhead costs for its implementation of GHSP.

Whenever possible (and always with proper systems in place to ensure accountability), low-income countries should also have more direct control of funds. Through MEPI and the HRH Program, local governments and/or academic institutions have been the primary recipients of funds and have been able to select the training partners whom they view as the best fit to address local priorities [15,16]. Definitive evidence that funding flexibility and allocation to local governments results in a more effective utilization of funds will be generated over the next few years as the initiatives described in this article undergo serial evaluations and audits.

### Competency-Based Training and Pedagogic Innovation

Training initiatives should prioritize the acquisition of competencies through sustained mentorship and supervision rather than the acquisition of knowledge through ad hoc, short-term lectures and seminars [4–7,30]. The competencies and skills that allow different cadres of health professionals to work together as a team and address both biomedical and psychosocial determinants of health are especially important and have begun to be prioritized only recently, such as in programs recently launched in Ethiopia and South Africa [4,9,31–33].

Given this commitment to sustained mentorship and supervision, MEPI, the HRH Program, and GHSP have all established “teaching” hospitals, health centers, and communities where trainees can witness firsthand how high-quality health care is delivered and good governance pursued across all levels of the health system [15,24,34]. To increase retention after graduation, the curricula and training materials developed through MEPI in 12 sub-Saharan African countries are specifically designed to prepare trainees for practice in remote and under-resourced health facilities [34,35]. Similarly, the GHSP has prioritized the acquisition of competencies and skills by physicians and nurses in rural areas [21]. Uniquely among the other initiatives described in this article, the HRH Program has also prioritized training of hospital administrators and implementation experts tasked with managing Rwanda’s health facilities and strengthening the health system [15]. The potential of information technology is being harnessed not only to overcome the shortage of faculty but also to drive pedagogical innovation through e-learning and reverse classroom approaches [9,23,29,36].

### Institutional Capacity Building

Development and training partners should be tasked with building institutional capacity within academic institutions and the public health sector of low-income countries and avoid the establishment of parallel systems [4,5,14].

MEPI, NEPI, the HRH Program, and GHSP are strengthening the teaching and mentoring skills of local faculty through a variety of approaches [4,7,13,14,21,26]. For example, in Kenya, MEPI has recruited and trained clinicians in rural areas to serve as adjunct faculty for trainees during their community-based rotations [37]. NEPI offers scholarships to candidates pursuing a master’s or doctorate degree in nursing and midwifery education [23]. The HRH Program and GHSP are twinning local faculty and trainees with US faculty based in the country to develop both curricula and training materials, as well as to drive pedagogical innovation [15,21]. Building research and evaluation capacity to answer research questions that are locally relevant may also help improve faculty retention. Thus, MEPI, NEPI, and the HRH Program seek to strengthen research and evaluation skills among local faculty, with a particular focus on implementation science [15,23,38].

### Sustainability Strategy

The engagement of development and training partners in low-income countries should neither end abruptly nor last indefinitely [13–15,26]. Funding and training expertise should gradually decrease over time until (and only when) both become no longer necessary. Conversely, local governments should assume responsibility for sustaining and further expanding these initiatives and have a long-term plan for hiring and adequately compensating the newly trained health professionals [4,7].

For example, in the HRH Program, faculty deployment by US academic institutions and funding from the US Centers for Disease Control (CDC) and the Global Fund will continue but gradually decrease from over 100% to 0% over the course of eight years, the time estimated for the training targets identified by the public health sector to be met [15]. Similarly, US faculty deployed through the GHSP are expected to serve as “force multipliers” by training local clinicians to serve as faculty and as “force multipliers” themselves, thus exponentially increasing the pool of experienced and committed educators over the years.

### Promoting Long-Lasting Partnerships and Communities of Practice

Academic institutions in low-income countries are seldom able to systematically collaborate on research and training initiatives with one another and with foreign academic institutions from high-income countries. Simultaneously, foreign academic institutions from high-income countries have resources and expertise that can benefit low-income countries while facing a growing demand from their own faculty and trainees to practice overseas [4,7,39,40].

By twinning local faculty with US faculty, the HRH Program and GHSP seek to foster academic collaborations that ideally will continue even after both initiatives have formally ended. Similarly, MEPI and NEPI have established communities of practice and advisory groups that allow faculty and academic institutions from many sub-Saharan African countries to collaborate among themselves and with their counterparts in the US through regular site visits, annual symposia, webinars, and joint academic writing [41].

## Challenges to the New Framework

There are many challenges associated with implementing ambitious and innovative health professional training initiatives such as the four described in this article. Primarily, we have found that establishing consensus among multiple stakeholders has been one of the most difficult and time-intensive steps, requiring extensive negotiations and leadership on all sides, as well as a shared commitment to the ultimate vision.

Additionally, the stakeholders initially lacked the resources, knowledge, or processes required to effectively initiate and sustain implementation. For instance, new policies developed for building research capacity (e.g., ethical approval and authorship protocol), training (e.g., accreditation and intellectual property for newly developed curricula), and health service delivery (e.g., credentialing, licensing, and malpractice coverage for foreign clinicians) have stretched significantly the bureaucracies of both the sub-Saharan and US academic institutions involved. Despite a concerted effort to build local administrative capacity, a large influx of US educators, researchers, and clinicians in the host countries has added a considerable amount of work on an already scarce and overstretched local staff. Reconciling differences in culture and practice between local and US staff has required extensive orientation trainings and ongoing reassessment of interpersonal and inter-institutional relationships. Lastly, monitoring and evaluation for the four initiatives is ongoing but at an early stage. Because of the scale and complexity of each initiative, implementation took precedence over monitoring and evaluation. Only recently has newly generated knowledge begun to inform implementation and further consolidate the new framework and best practices outlined in this article. Evidence to date, albeit in its infancy, has been encouraging that programs with this approach represent a “paradigm shift” for global health education [42].

## Conclusion

A sizeable, diverse, and competent needs-based health workforce is essential to strengthen health systems in low-income countries, which suffer from a severe shortage of health professionals. The harsh consequences of this shortage in West Africa, for example, demonstrates the urgency to fund and develop innovative partnerships to bolster smart training initiatives moving forward. In this article, we outline a new framework for health professional training initiatives informed by the best practices adopted by four innovative US-led health professional training initiatives, which prioritize country ownership, funding flexibility, the acquisition of competencies, institutional capacity building, and long-term sustainability. Though there are notable challenges that exist in operationalizing these best practices, we believe that greater investments into future programs that adopt this framework holds great promise to meaningfully address the workforce shortage that has plagued the poorest countries in the world for too long.

## Abbreviations

CDC: Centers for Disease Control
GHSP: Global Health Service Partnership
HRH: Rwanda Human Resources for Health
MEPI: Medical Education Training Partnership Initiative
NEPI: Nursing Training Partnership Initiative
NGO: non-governmental organization
PEPFAR: US President’s Emergency Plan for AIDS Relief
USAID: US Agency for International Development

## References

[1] World Health Organization. The world health report: 2006: working together for health Geneva: World Health Organization; 2006 http://www.who.int/iris/handle/10665/43432. Accessed 12 May 2015.

[2] Chen L, Evans T, Anand S, Boufford JI, Brown H, Chowdhury M, et al Human resources for health: overcoming the crisis. The Lancet. 2004;364: 1984–1990. 10.1016/S0140-6736(04)17482-5 15567015

[3] Hongoro C, McPake B. How to bridge the gap in human resources for health. The Lancet. 2004;364: 1451–1456. 15488222 10.1016/S0140-6736(04)17229-2

[4] Frenk J, Chen L, Bhutta ZA, Cohen J, Crisp N, Evans T, et al Health professionals for a new century: transforming education to strengthen health systems in an interdependent world. The Lancet. 376: 1923–1958. 10.1016/S0140-6736(10)61854-5 21112623

[5] Celletti F, Reynolds TA, Wright A, Stoertz A, Dayrit M. Educating a New Generation of Doctors to Improve the Health of Populations in Low- and Middle-Income Countries. PLoS Med. 2011;8: e1001108 10.1371/journal.pmed.1001108 22028631 PMC3196469

[6] Mullan F, Frehywot S, Omaswa F, Buch E, Chen C, Greysen SR, et al Medical schools in sub-Saharan Africa. The Lancet. 2011;377: 1113–1121. 10.1016/S0140-6736(10)61961-7 21074256

[7] Kolars JC, Cahill K, Donkor P, Kaaya E, Lawson A, Serwadda D, et al Perspective: Partnering for Medical Education in Sub-Saharan Africa. Acad Med. 2012;87: 216–220. 10.1097/ACM.0b013e31823ede39 22189887

[8] Willis-Shattuck M, Bidwell P, Thomas S, Wyness L, Blaauw D, Ditlopo P. Motivation and retention of health workers in developing countries: a systematic review. BMC Health Serv Res. 2008;8: 247 10.1186/1472-6963-8-247 19055827 PMC2612662

[9] Crisp N, Chen L. Global Supply of Health Professionals. N Engl J Med. 2014;370: 950–957. 10.1056/NEJMra1111610 24597868

[10] Crane J. Scrambling for Africa? Universities and global health. The Lancet. 377: 1388–1390. 10.1016/S0140-6736(10)61920-4 21074254

[11] Merson MH. University Engagement in Global Health. N Engl J Med. 2014;370: 1676–1678. 10.1056/NEJMp1401124 24785204

[12] Frenk J, Moon S. Governance Challenges in Global Health. N Engl J Med. 2013;368: 936–942. 10.1056/NEJMra1109339 23465103

[13] World Health Organization Maximizing Positive Synergies Collaborative Group, Samb B, Evans T, Dybul M, Atun R, Moatti J-P, et al An assessment of interactions between global health initiatives and country health systems. Lancet. 2009;373: 2137–2169. 10.1016/S0140-6736(09)60919-3 19541040

[14] Vujicic M, Weber SE, Nikolic IA, Atun R, Kumar R. An analysis of GAVI, the Global Fund and World Bank support for human resources for health in developing countries. Health Policy Plan. 2012;27: 649–657. 10.1093/heapol/czs012 22333685

[15] Binagwaho A, Kyamanywa P, Farmer PE, Nuthulaganti T, Umubyeyi B, Nyemazi JP, et al The Human Resources for Health Program in Rwanda—A New Partnership. N Engl J Med. 2013;369: 2054–2059. 10.1056/NEJMsr1302176 24256385

[16] Farmer PE, Nutt CT, Wagner CM, Sekabaraga C, Nuthulaganti T, Weigel JL, et al Reduced premature mortality in Rwanda: lessons from success. BMJ. 2013;346: f65–f65. 10.1136/bmj.f65 23335479 PMC3548616

[17] Asante AD, Negin J, Hall J, Dewdney J, Zwi AB. Analysis of policy implications and challenges of the Cuban health assistance program related to human resources for health in the Pacific. Hum Resour Health. 2012;10: 10 22558940 10.1186/1478-4491-10-10 PMC3447691

[18] University of Oulu. MEDUNAM. http://www.medunam.net/. Accessed 8 Mar 2015.

[19] Goosby E, Von Zinkernagel D, Holmes C, Haroz D, Walsh T. Raising the bar: PEPFAR and new paradigms for global health. J Acquir Immune Defic Syndr 1999. 2012;60 Suppl 3: S158–162. 10.1097/QAI.0b013e31825d057c 22797738

[20] Collins FS, Glass RI, Whitescarver J, Wakefield M, Goosby EP. Public health. Developing health workforce capacity in Africa. Science. 2010;330: 1324–1325. 10.1126/science.1199930 21127233 PMC5101931

[21] Kerry VB, Mullan F. Global Health Service Partnership: building health professional leadership. Lancet. 2014;383: 1688–1691. 10.1016/S0140-6736(13)61683-9 24360618

[22] OECD. The Paris Declaration on Aid Effectiveness. In: OECD iLibrary. http://www.oecd.org/dac/effectiveness/parisdeclarationandaccraagendaforaction.htm. Accessed 10 Mar 2015.

[23] Middleton L, Howard AA, Dohrn J, Von Zinkernagel D, Parham Hopson D, Aranda-Naranjo B, et al The Nursing Education Partnership Initiative (NEPI): Innovations in Nursing and Midwifery Education. Acad Med. 2014;89: S24–S28. 10.1097/ACM.0000000000000342 25072571

[24] Olapade-Olaopa EO, Baird S, Kiguli-Malwadde E, Kolars JC. Growing Partnerships: Leveraging the Power of Collaboration Through the Medical Education Partnership Initiative. Acad Med. 2014;89: S19–S23. 10.1097/ACM.0000000000000345 25072570

[25] Goosby EP, von Zinkernagel D. The Medical and Nursing Education Partnership Initiatives: Acad Med. 2014;89: S5–S7. 10.1097/ACM.0000000000000346 25072578 PMC4327847

[26] The Global Fund to fight AIDS, Tuberculosis and Malaria. Global Fund Strategy 2012–2016. http://www.theglobalfund.org/en/about/strategy/. Accessed 23 Dec 2014.

[27] Walton DA, Farmer PE, Lambert W, Léandre F, Koenig SP, Mukherjee JS. Integrated HIV Prevention and Care Strengthens Primary Health Care: Lessons from Rural Haiti. J Public Health Policy. 2004;25: 137–158. 15255381 10.1057/palgrave.jphp.3190013

[28] Price JE, Leslie JA, Welsh M, Binagwaho A. Integrating HIV clinical services into primary health care in Rwanda: a measure of quantitative effects. AIDS Care. 2009;21: 608–614. 10.1080/09540120802310957 19444669

[29] Lisasi E, Kulanga A, Muiruri C, Killewo L, Fadhili N, Mimano L, et al Modernizing and Transforming Medical Education at the Kilimanjaro Christian Medical University College: Acad Med. 2014;89: S60–S64. 10.1097/ACM.0000000000000327 25072581 PMC4115810

[30] Cancedda C, Farmer PE, Kyamanywa P, Riviello R, Rhatigan J, Wagner CM, et al Enhancing Formal Educational and In-Service Training Programs in Rural Rwanda: A Partnership Among the Public Sector, a Nongovernmental Organization, and Academia. Acad Med. 2014;89: 1117–1124. 10.1097/ACM.0000000000000376 24979292

[31] Girma S, Yohannes AG, Kitaw Y, Ye-Ebiyo Y, Seyoum A, Desta H, et al Human Resource Development for Health in Ethiopia: Challenges of Achieving the Millennium development Goals. Ethiop J Health Dev. 2008;21.

[32] Lehmann U. Strengthening human resources for Primary Health Care: Primary Health Care: systems support. South Afr Health Rev. 2008; 163–177.

[33] Teklehaimanot HD, Teklehaimanot A. Human resource development for a community-based health extension program: a case study from Ethiopia. Hum Resour Health. 2013;11: 39 10.1186/1478-4491-11-39 23961920 PMC3751859

[34] Mariam DH, Sagay AS, Arubaku W, Bailey RJ, Baingana RK, Burani A, et al Community-Based Education Programs in Africa: Faculty Experience Within the Medical Education Partnership Initiative (MEPI) Network. Acad Med. 2014;89: S50–S54. 10.1097/ACM.0000000000000330 25072579

[35] Derbew M, Animut N, Talib ZM, Mehtsun S, Hamburger EK. Ethiopian Medical Schools’ Rapid Scale-up to Support the Government’s Goal of Universal Coverage: Acad Med. 2014;89: S40–S44. 10.1097/ACM.0000000000000326 25072576

[36] Frehywot S, Mullan F, Vovides Y, Korhumel K, Chale SB, Infanzon A, et al Building Communities of Practice: MEPI Creates a Commons. Acad Med. 2014;89: S45–S49. 10.1097/ACM.0000000000000349 25072577

[37] Child MJ, Kiarie JN, Allen SM, Nduati R, Wasserheit JN, Kibore MW, et al Expanding Clinical Medical Training Opportunities at the University of Nairobi: Adapting a Regional Medical Education Model From the WWAMI Program at the University of Washington. Acad Med. 2014;89: S35–S39. 10.1097/ACM.0000000000000350 25072575 PMC4183931

[38] Glass RI, Razak MH, Said M. The Importance of Research in the MEPI Program: Perspectives From the National Institutes of Health. Acad Med. 2014;89: S9–S10. 10.1097/ACM.0000000000000351 25072589 PMC4118423

[39] Kerry VB, Ndung’u T, Walensky RP, Lee PT, Kayanja VFIB, Bangsberg DR. Managing the Demand for Global Health Education. PLoS Med. 2011;8: e1001118 10.1371/journal.pmed.1001118 22087076 PMC3210750

[40] Syed SB, Dadwal V, Rutter P, Storr J, Hightower JD, Gooden R, et al Developed-developing country partnerships: benefits to developed countries. Glob Health. 2012;8 10.5339/gcsp.2012.8 22709651 PMC3459713

[41] Binagwaho A, Nutt CT, Mutabazi V, Karema C, Nsanzimana S, Gasana M, et al Shared learning in an interconnected world: innovations to advance global health equity. Glob Health. 2014;9: 37. 10.1186/1744-8603-9-37 PMC3765795 24119388

[42] Talib ZM, Kiguli-Malwadde E, Wohltjen H, Derbew M, Mulla Y, Olaleye D, et al Transforming health professions’ education through in-country collaboration: examining the consortia among African medical schools catalyzed by the Medical Education Partnership Initiative. Hum Resour Health. 2015;13: 1 10.1186/1478-4491-13-1 25588887 PMC4355474

